# Characterising heart rhythm abnormalities associated with Xp22.31 deletion

**DOI:** 10.1136/jmg-2022-108862

**Published:** 2022-11-15

**Authors:** Georgina Wren, Emily Baker, Jack Underwood, Trevor Humby, Andrew Thompson, George Kirov, Valentina Escott-Price, William Davies

**Affiliations:** 1 School of Psychology, Cardiff University, Cardiff, UK; 2 Dementia Research Institute, Cardiff University, Cardiff, UK; 3 MRC Centre for Neuropsychiatric Genetics and Genomics and Division of Psychological Medicine and Clinical Neurosciences, School of Medicine, Cardiff University, Cardiff, UK; 4 Neuroscience and Mental Health Innovation Institute, Cardiff University, Cardiff, UK; 5 Cardiff and Vale University Health Board, University Hospital of Wales, Cardiff, UK

**Keywords:** Anemia, Arrhythmias, Cardiac, Dermatology, Gastrointestinal Diseases, Genetic Association Studies

## Abstract

**Background:**

Genetic deletions at Xp22.31 are associated with the skin condition X linked ichthyosis (XLI), and with a substantially increased risk of atrial fibrillation/flutter (AF), in males. AF is associated with elevated thrombosis, heart failure, stroke and dementia risk.

**Methods:**

Through: (a) examining deletion carriers with a diagnosis of AF in UK Biobank, (b) undertaking an online survey regarding abnormal heart rhythms (AHRs) in men/boys with XLI and female carriers of XLI-associated deletions and (c) screening for association between common genetic variants within Xp22.31 and idiopathic AF-related conditions in UK Biobank, we have investigated how AHRs manifest in deletion carriers, and have identified associated risk factors/comorbidities and candidate gene(s). Finally, we examined attitudes towards heart screening in deletion carriers.

**Results:**

We show that AHRs may affect up to 35% of deletion carriers (compared with <20% of age-matched non-carriers), show no consistent pattern of onset but may be precipitated by stress, and typically resolve quickly and respond well to intervention. Gastrointestinal (GI) conditions and asthma/anaemia were the most strongly associated comorbidities in male and female deletion carriers with AHR, respectively. Genetic analysis indicated significant enrichment of common AF risk variants around *STS* (7 065 298–7 272 682 bp in GRCh37/hg19 genome build) in males, and of common GI disorder and asthma/anaemia risk variants around *PNPLA4* (7 866 804–7 895 780 bp) in males and females, respectively. Deletion carriers were overwhelmingly in favour of cardiac screening implementation.

**Conclusion:**

Our data suggest AHRs are frequently associated with Xp22.31 deletion, and highlight subgroups of deletion carriers that may be prioritised for screening. Examining cardiac function further in deletion carriers, and in model systems lacking steroid sulfatase, may clarify AF pathophysiology.

**WHAT IS ALREADY KNOWN ON THIS TOPIC**

- Deletions at Xp22.31 are associated with a substantially increased likelihood of diagnosis of atrial fibrillation/flutter in middle-aged individuals, but how abnormal heart rhythms (AHRs) manifest in this population is unclear.

**WHAT THIS STUDY ADDS**

- We show that AHR in Xp22.31 deletion carriers are common, comparatively mild, largely responsive to treatment, tend to be precipitated by stress, and are comorbid with gastrointestinal conditions, asthma and anaemia. We identify *STS* as a candidate gene for AHR and *PNPLA4* as a candidate gene for comorbidities. We also show that deletion carriers are in favour of cardiac screening.

**HOW THIS STUDY MIGHT AFFECT RESEARCH, PRACTICE OR POLICY**

- The study guides future work in Xp22.31 deletion carriers and suggests that work in steroid sulfatase-deficient model systems may provide insights into the pathophysiology of atrial fibrillation. The feasibility of cardiac screening in individuals carrying Xp22.31 deletions or diagnosed with X linked ichthyosis (particularly those exhibiting gastrointestinal conditions, asthma or anaemia) might be investigated.

## Introduction

Prenatal screening estimates indicate that genetic deletions within Xp22.31 occur in around 1 in 1500 males and 1 in 750 females.^1 2^ Twenty-five per cent to 60% of males carrying such variants are diagnosed with X linked ichthyosis (XLI (MIM: 308100)), a dermatological condition characterised by skin scaling and resulting from absence of the steroid sulfatase (STS) enzyme.^3^ In males, typically sized XLI deletions of 1.5–1.7 Mb are associated with extracutaneous symptoms including: corneal opacities,^3^ testicular maldescent,^3^ focal epilepsy,^4^ atopic disorders (asthma, eczema and allergic rhinitis),^5–7^ aberrant haemostasis and fibrosis,^8^ and increased neurodevelopmental and mood diagnoses/traits^9^; larger deletions (>2.5 Mb) can be associated with more severe developmental issues. Heterozygous female carriers can also show high neurodevelopmental and mood traits, and around 60% experience delayed or prolonged labour during childbirth, but these individuals do not usually exhibit the physical (skin, eye and neurological) phenotypes seen in males.^10^

Using the large UK Biobank sample recruited from the general population of the UK, we have recently shown that middle-aged men carrying deletions of 0.8–2.5 Mb within Xp22.31 (a region which escapes X-inactivation in females^11^) are at significantly elevated (approximately fourfold) risk of being diagnosed with atrial fibrillation and/or atrial flutter (AF) (but not other cardiovascular or metabolic conditions) compared with male non-carriers^12^; the excess of AF diagnoses was not apparent in female deletion carriers,^12^ nor was it apparent in males with genetic duplication of the same region.^13^ AF is the most common supraventricular arrhythmia, and is characterised by dysregulated and chaotic twitching of the atrium.^14^ The severity of the condition can vary from a single, rapidly resolving incident, to recurrent, rapidly resolving episodes (‘paroxysmal AF’), episodes resolving more slowly (‘persistent AF’) or not resolving at all (‘permanent AF’).^15^ Xp22.31 deletions could potentially explain up to 1 in 300 cases of AF in middle-aged men.^15^

AF can have significant effects on morbidity and mortality with downstream consequences including: thrombosis due to turbulent cardiac blood flow, embolism and stroke, as well as dementia.^16^ Risk factors for idiopathic AF include: age and heart tissue pathology (eg, arising due to hypertension, valvular or congenital heart disease, pericarditis, cardiomyopathy and diabetes), and the condition may be secondary to pulmonary conditions such as asthma.^14^ Recent case reports in males with XLI have described paroxysmal supraventricular tachycardia with anaemia,^17^ and AF with kidney disease, type 2 diabetes mellitus, hypertension and dyslipidaemia.^18^ AF is commonly associated with the sensation of an irregular or very fast heartbeat even while at rest, with palpitations and chest pain, and with breathlessness, fatigue and feelings of dizziness or faintness; however, in some cases, AF is asymptomatic and may be picked up incidentally during medical examinations or check-ups.^15^ AF may be screened for, and diagnosed in, ‘at-risk’ individuals through a combination of ECG and echocardiograms, chest X-ray and blood tests.^15^

The purpose of the present study was fourfold. First, we aimed to identify comorbidities in middle-aged male and female carriers of XLI-associated deletions in the UK Biobank who had been diagnosed with AF. Second, we aimed to characterise abnormal heart rhythm (AHR)-related phenotypes and comorbidities in men and boys with XLI, and in female carriers, through an online survey. These initial analyses were intended to enhance our understanding of the nature and course of, and risk factors for, AF in individuals with Xp22.31 deletions with a view to improving AF prediction within this genetic cohort. Convergent findings across our two distinct participant groups (UK Biobank and online samples) would be expected to be robust and generalisable. Third, we aimed to gauge opinion on screening for cardiac abnormalities in patients with confirmed XLI/Xp22.31 deletion. Finally, we aimed to identify candidate genes within the Xp22.31 deletion interval by screening for common risk variants for idiopathic AF and associated conditions in the UK Biobank sample; this would provide clues regarding biological mechanisms for any phenotype–genotype associations and would suggest future studies geared towards clarifying these.

## Methods

### Comorbid conditions in carriers of Xp22.31 deletions and AF in UK Biobank sample

The UK Biobank sample comprises almost half a million participants recruited between 2006 and 2010 at ages 40–69 years from the UK general population for which anonymised genotype and extensive phenotype data are available.^19^ Descriptive International Classification of Diseases 10th Revision (ICD-10) codes and self-reported ‘blistering/desquamating disorder’ (ie, ichthyosis) diagnoses in Xp22.31 deletion carriers (0.8–2.5 Mb around *STS*) from the UK Biobank sample were compared between individuals diagnosed with AF and those not diagnosed with AF.

### Online survey

#### Participants

Adult (>18 years) men with a confirmed diagnosis of XLI, confirmed adult female carriers and parents of boys with a confirmed diagnosis of XLI were recruited via relevant charities, online patient support groups and social media; diagnosis/carrier status was typically confirmed on the basis of a combination of: family history, assessment of skin condition and biochemical/genetic testing. Participants were directed to an online survey which was open from 2 September to 22 December 2021, and anonymised data were returned to the study team upon completion.

#### Survey structure

The survey was designed in Qualtrics^20^ and was available via a specific URL. Participants were initially asked to provide basic demographic information including their (or their son’s) age, country of residence and ethnicity, before confirming the basis of their (or their son’s) diagnosis/carrier status. Men with XLI, or parents of boys with XLI, were then asked to rate their (or their son’s) skin severity across life based on the Congenital Ichthyoses Severity Index (possible scores 2–8),^21^ and check whether their son(s) had been affected by any of the following developmental conditions: testicular maldescent or a neurodevelopmental/neurological condition. Participants were then asked to specify if they (or their sons) had ever experienced, or been diagnosed by a medical professional with, an AHR (and if so, to specify the condition); they were also asked to check whether they (or their sons) had been diagnosed with an array of other cardiovascular or metabolic conditions known to be risk factors/comorbidities for AF (online supplemental text 1), and to confirm whether or not there was a family history of cardiovascular issues. There were then a series of questions about the precipitants, nature, developmental course and severity of any self-reported heart arrhythmias and their treatments. Finally, participants were presented with a short vignette about the potential link between XLI and AF, and the possible complications associated with AF (online supplemental text 2) and were asked to rate the following statements on a 5-point scale ranging from strongly disagree to strongly agree: ‘Do you think that risk of AF in XLI males is a significant health concern?’, ‘Do you think that males should be screened for heart abnormalities routinely following confirmation of XLI?’, ‘I believe that screening for heart problems in XLI is a good use of healthcare funding’, ‘I would be happy to attend/bring my son to a doctor’s surgery/hospital regularly for heart screening’, ‘I believe that knowing that I/my son have a heart condition with possible adverse consequences is preferable to not knowing’, ‘I believe that the benefits associated with screening for, monitoring and treating any heart condition outweigh their risks’ and ‘I require more information about the relationship between XLI and heart conditions in order to make any judgement about the benefit of screening’. Participants could leave their email address if they wanted further information or were happy to be recontacted.

10.1136/jmg-2022-108862.supp1 Supplementary data

#### Survey analysis

For each group, the proportion of individuals reporting AHRs was calculated, and the nature of those abnormalities characterised. Subsequently, each participant group was divided into individuals with and without self-reported AHRs, and the two subgroups were then directly compared across demographic measures and medical phenotypes to identify factors co-segregating with heart arrhythmias within these populations. Continuous variables were compared between groups with unpaired t-test or Mann-Whitney U test depending on normality of the data, and categorical data were analysed by Χ^2^ or Fisher’s exact test; ORs are presented as a measure of effect size. Where multiple medical phenotypes were assessed, Benjamini-Hochberg False Discovery Rate correction was applied.^22^

### Molecular genetics in UK Biobank sample

A total of 363 693 white British and Irish individuals remained in our UK Biobank sample after removing related individuals and those who have since chosen to withdraw from the study. Diagnoses were coded according to ICD-10. The UK Biobank contains 3 917 799 imputed SNPs on the X chromosome. These imputed data were quality controlled (QC) by removing rare SNPs with minor allele frequency <0.01, SNPs imputed with poor accuracy (INFO <0.4) and SNPs with missing data proportion >0.05. The data were split into males and females. After these QC steps, 262 278 SNPs in 195 638 females and 267 937 SNPs in 168 055 males remained for analysis.

First, SNPs in the region of interest (ChrX:6 435 064–8 414 482 GRCh37/hg19 genome build, that is, consensus deletion interval for individuals with AF in UK Biobank) were extracted: 3966 and 4060 SNPs remain in females and males, respectively; individual SNPs with −log_10_(p)>4.90 (p<1.26×10^−5^) or −log_10_(p)>4.91 (p<1.23×10^–5^) were regarded as significant following multiple testing correction in females and males, respectively. We then ran multiple association analyses between all SNPs in the region of interest with a number of different phenotypes linked to AF. We investigated AF (ICD-10 code ‘I48’), stroke (ICD-10 code ‘I64’), acute myocardial infarction (ICD-10 code ‘I21’), dementia (combined ICD-10 codes ‘F00–03’), asthma (ICD-10 code ‘J45’), anaemia (combined ICD-10 codes ‘D50−52’) and gastrointestinal (GI) disorders (combined ICD-10 codes ‘K50−59’). The association analyses were run for each phenotype in males and females separately. Fifteen principal components, array and age were included in the model as covariates. Finally, we used MAGMA^23^ to run a gene-based analysis for the different association analyses to determine whether the SNPs within our region of interest had an aggregate effect in a particular protein-coding gene. The SNPs were annotated to genes using the gene location file from the MAGMA website; this contains gene locations from protein-coding genes obtained from the National Center for Biotechnology Information (NCBI) site. SNPs within the transcription start and stop sites were included. The mean Χ^2^ gene-based analysis was used on the summary statistics from the association analyses, and unadjusted p values are presented.

### Availability of data and materials

UK Biobank data are available upon application to that resource.^24^ Online survey data generated or analysed during this study are included in this published article and the online supplemental file 1, or are available from http://doi.org/10.17035/d.2022.0230251614.

## Results

### Comorbid conditions in carriers of Xp22.31 deletions diagnosed with AF in UK Biobank

Of the 86 male Xp22.31 deletion carriers previously identified in UK Biobank, 9 had been diagnosed with AF. These nine individuals, were on average, significantly older than the remaining 77 (age in 2016: 69.8±1.6 years vs 65.3±0.9 years, t[11.7]=2.40, p=0.034), but the two groups did not differ with respect to self-reported blistering/desquamating disorder diagnoses (1 of 9 (11%) vs 3 of 77 (4%), Fisher’s exact test p=0.36). In the nine subjects with AF, the most common comorbid diagnoses were: GI problems (viral intestinal infection in one individual, and non-infective gastroenteritis and colitis in two individuals) and respiratory conditions (asthma in one individual and pneumonia in two individuals). Comparison of the most common diagnoses in the male deletion carrier AF versus non-AF group revealed that only the prevalence of ‘non-infective gastroenteritis and colitis’ differed significantly between groups, being more common in the former group (2 of 9 (22%) vs 1 of 77 (1%), p=0.028 Fisher’s exact test). Of the 312 female deletion carriers identified in the UK Biobank, just 3 had been diagnosed with AF. All three carriers diagnosed with AF displayed prominent cardiovascular abnormalities and had also been diagnosed with left ventricular failure. The other most common comorbidities in female carriers with AF were dyspnoea (two individuals) and bone/joint conditions (rheumatoid arthritis in one patient and fracture in the second); one individual diagnosed with AF presented with acute renal failure and anaemia.

### Online survey data

#### Comparison of demographics and comorbidities in individuals with and without AHR

We recruited a total of 191 participants (43 adult men with XLI, 79 female carriers and 69 boys with XLI), although not all participants completed all aspects of the survey. The prevalence of self (or parent)-reported AHRs was similar across the three groups (35% in adult men with XLI, 28% in adult female carriers and 28% in boys with XLI); across the three groups, individuals affected by AHRs and those unaffected did not differ significantly with respect to age, country of residence and ethnicity, although individuals experiencing AHRs tended to be older on average (online supplemental table 1). Across both male groups combined, severity of the skin condition did not differ between those with AHR and those without (4.1±1.7 and 3.7±1.4, respectively, U=1092.0, z=−0.132, p=0.90). Boys with AHR did not exhibit more developmental conditions than those without (testicular maldescent: 20% vs 10%, respectively, p=0.09; neurodevelopmental disorder: 27% vs 27%, respectively, p=1.0). Consistent with a possible genetic influence on arrhythmia risk, males self-reporting AHRs were more likely to endorse a family history of cardiovascular issues than males not reporting (77% vs 46%, respectively, p=0.005); this pattern of results was maintained when female carriers were also included in the analysis (74% vs 49%, respectively, χ^2^_(1)_=8.88, p=0.003).

Of all the comorbid medical conditions assessed, only ‘gut problem’ was significantly more frequently self-reported in males with AHR than males without (OR: 7.0; 95% CI: 1.3 to 38.4, p=0.022). ‘Heart murmur’ (OR >10, p=0.005), anaemia (OR: 5.0; 95% CI: 1.5 to 17.2, p=0.011) and asthma (OR: 5.0; 95% CI: 1.3 to 18.2, p=0.017) were all significantly more common in female carriers reporting heart rhythm abnormalities than in those without. When adult/young male, and female deletion-carrying groups were all combined, four medical conditions were significantly more common in AHR than in non-AHR groups: ‘heart valve disease or malformation’ (OR: 10.6 (95% CI: 1.2 to 97.4), p=0.025), ‘anaemia’ (OR: 3.2 (95% CI: 1.1 to 8.7), p=0.027), ‘asthma’ (OR: 2.9 (95% CI: 1.3 to 6.4), p=0.010) and ‘gut problem’ (OR: 4.9 (95% CI: 1.7 to 14.3), p=0.004); of these, only ‘gut problem’ survived Benjamini-Hochberg False Discovery Rate correction (adjusted p=0.1) (online supplemental table 2A, B).

#### Nature of rhythm abnormalities in individuals with AHR

In adult men, AF had been diagnosed by a medical professional in 60% of individuals with AHR, and tachycardia in 27%. Thirty per cent of female carriers with AHR had been diagnosed with AF, and 60% with tachycardia. In boys with XLI and AHR, 53% had been diagnosed with tachycardia, 40% with bradycardia and 20% with AF.

Stress was cited in the top three precipitating factors for AHR across all three groups (referred to as a precipitant by 42% of men with XLI, 45% of female carriers and 14% of parents of boys with XLI); other precipitants were less-consistently identified but included ‘no obvious cause’ (men and boys with XLI), ‘exercise’ (female carriers) and ‘increased body temperature’ (boys with XLI) (online supplemental table 3). There was no clear pattern as to when AHRs onset during the day across all three groups (online supplemental table 4). For the majority of participants who provided responses, AHRs resolved spontaneously within 12 hours (82% of men with XLI, 85% female carriers and 82% boys with XLI), and often within 1 hour (36%, 61% and 55%, respectively). Where interventional strategies (typically breathing exercises) were required, their success was reported as being good (>5 out of 10) in 61% of males with XLI, in 100% of female carriers and in 86% of boys with XLI.

#### Response to the vignette

**Figure 1:**
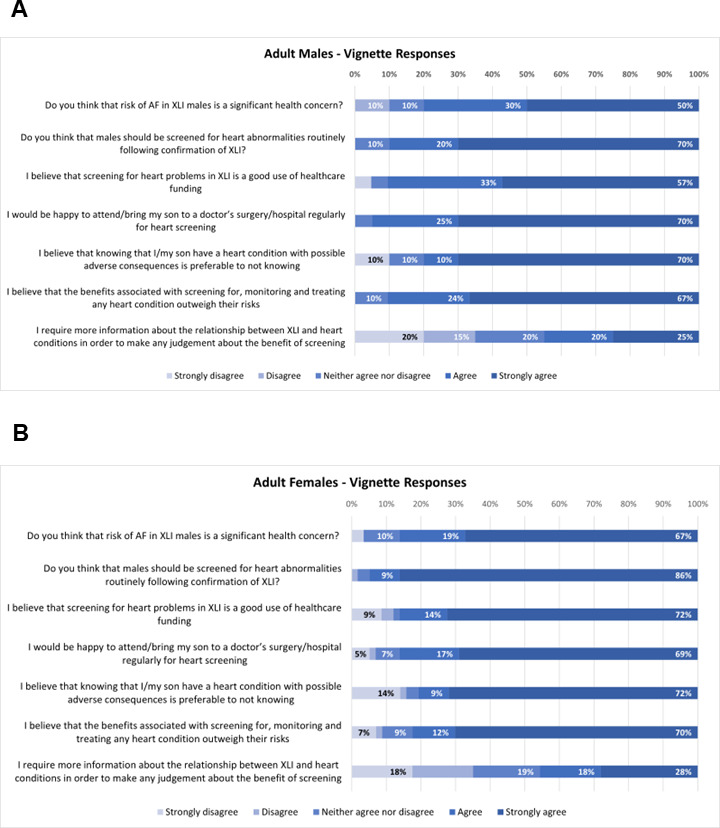
Responses to the vignette in adult men with XLI (n=21) (A) and adult female carriers (n=58) (B). AF, atrial fibrillation/flutter; XLI, X linked ichthyosis.

A total of ≥80% of men with XLI (figure 1A) and female carriers (figure 1B) agreed or strongly agreed that: (1) risk of AF in XLI was a significant health concern, (2) cardiac screening should be routinely performed following confirmation of XLI, (3) screening for heart problems in XLI is a good use of healthcare funding, (4) they would be happy attending, or bringing their child to attend, heart screening appointments, (5) they would prefer to be made aware of potentially adverse medical conditions to not know, and (6) the potential benefits of heart screening outweighed the risks. Fewer than 50% of individuals from these two groups stated that they required further information regarding the link between XLI and heart arrhythmias.

### Molecular genetic analyses

**Figure 2:**
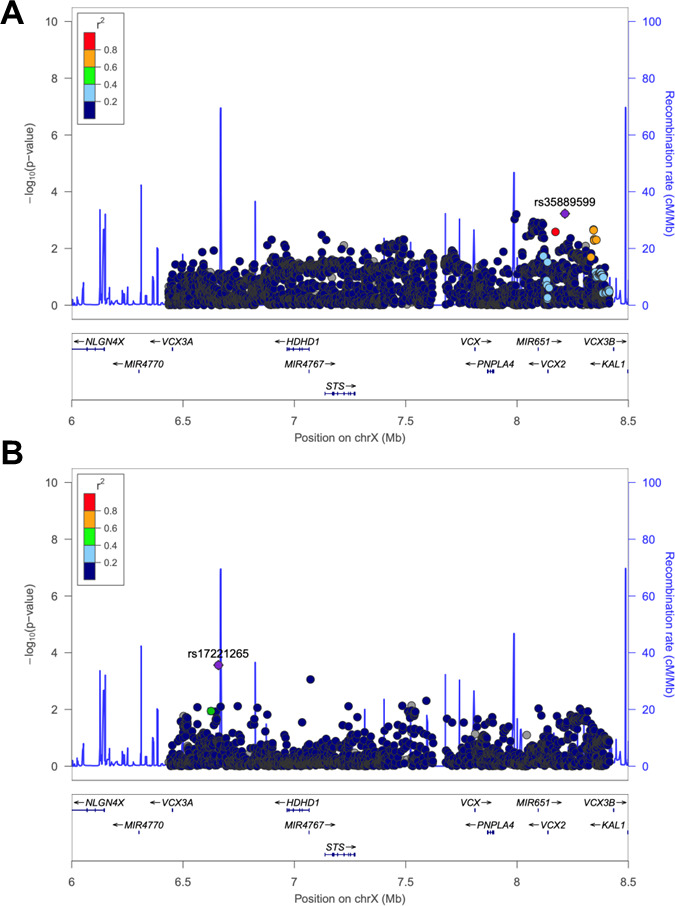
LocusZoom plots showing the SNPs most significantly associated with atrial fibrillation/flutter in the Xp22.31 deletion consensus interval in males (A) and females (B).

Across all conditions, only two closely linked individual SNPs (rs141750978;chrX:7 934 924 and rs2051996;chrX:7 933 380 GRCh37/h19 genome build) exceeded the threshold for significant association (p=2.24×10^−6^ and 2.39×10^−6^, respectively), and this was with GI disorders in males (42 680 cases (25.4%) vs 125 375 controls (74.6%)). These SNPs are located closest to the *PNPLA4* transcriptional start site^25^ and show evidence for gene expression correlation with *PNPLA4* in small intestine terminal ileum (effect size 0.15, p=0.009).^26^ Gene-based analysis suggested a nominally significant association between *STS* (chrX:7 065 298–7 272 682, GRCh37/h19 genome build) and AF in males (4556 cases (2.7%) vs 163 499 controls (97.3%), p=0.041) but not in females (2144 cases (1.1%) vs 193 494 controls (98.9%), p=0.793) (table 1 and figure 2). No significant gene-based associations were identified in either males or females for stroke, acute myocardial infarction or dementia. Nominally significant associations between *PNPLA4* and asthma (p=0.040) and anaemia (p=0.013) were noted in females only. Finally, gene-based analysis suggested significant associations between *VCX3A*, *VCX*, *PNPLA4* and *VCX2* genes and GI conditions in males only (p values between 0.018 and 0.044). Gene-based analysis results for medical conditions related to AF are presented in online supplemental tables 5–10.

**Table 1:** A gene-based analysis of SNPs associated with atrial fibrillation/flutter in the consensus Xp22.31 deletion interval in males (4556 cases vs 163 499 controls) and females (2144 cases vs 193 494 controls) from the UK Biobank

Gene	Start (bp)	Stop (bp)	Males			Females		
			NSNPs	NParam	P value	NSNPs	NParam	P value
*VCX3A*	6 451 659	6 453 159	5	3	0.229	5	3	0.745
*HDHD1*	6 966 961	7 066 231	225	12	0.061	223	13	0.983
*STS*	7 065 298	7 272 682	343	20	0.041*	336	20	0.793
*VCX*	7 810 303	7 812 184	4	2	0.639	3	2	0.259
*PNPLA4*	7 866 804	7 895 780	39	6	0.633	39	6	0.278
*VCX2*	8 137 985	8 139 308	11	4	0.444	8	3	0.444

*P<0.05.

NParam, the number of relevant parameters used in the model (essentially the number of independent SNPs in the gene); NSNPs, the number of SNPs in the data annotated to the gene.

## Discussion

Using the UK Biobank sample, we previously showed that approximately 10% of middle-aged men carrying Xp22.31 deletions had been diagnosed with AF, a prevalence around four times than that in the non-carrier male population.^12^ Theoretically, the increased prevalence of AF in male Xp22.31 deletion carriers might have been due to the administration of retinoid-derived medications sometimes used to treat severe skin scaling which can have extensive side-effect profiles.^27 28^ However, <5% of male Xp22.31 deletion carriers within UK Biobank reported being formally diagnosed with ichthyosis, AHRs are not typically reported as a side effect of acitretin/(iso)tretinoin administration,^27 29^ and no male deletion carriers within the UK Biobank sample self-reported receiving acitretin or (iso)tretinoin treatment. Therefore, it is likely that the Xp22.31 deletion somehow confers a biological predisposition to AF. In the present study, we aimed to characterise the features and causes of cardiac rhythm abnormalities associated with this genetic variant more fully. We also aimed to investigate the acceptability of cardiac screening for deletion carriers.

Our online survey suggested self-reported AHRs in 28%–35% of male and female carriers of Xp22.31 deletions, and, consistent with this, many affected carriers had received a cardiac arrhythmia-related diagnosis from a medical professional and reported a family history of cardiovascular issues. This prevalence figure is likely higher than the 10% figure reported previously as it includes multiple causes of AHR in addition to AF and may include subclinical and undiagnosed cases; on the one hand, true prevalence rates may be underestimated given that only perceptible and symptomatic AHRs can be self-reported, but on the other hand, reported rates may be somewhat overinflated due to response bias. While we acknowledge the limitations of self-reported data on AHRs (eg, possible misreporting by participants and challenges associated with parental reporting on their sons’ experiences), this assessment method allows interrogation of comparatively large samples and provides information on AHRs occurring at time points across the lifespan as opposed to temporally restricted clinical monitoring via, for example, ECG. While AHR prevalence rates across the three online samples appear broadly similar, we note that the expected pattern of data was obtained, that is, highest rate (and worst response to intervention) in older, hemizygous males and lower rates (and better intervention responses) in heterozygous females and younger males. The prevalence of AHRs in our deletion-carrying population appears elevated compared with that in non-carrier samples, for example, arrhythmia was self-reported in 13.3% of men and 21.9% of women aged 40–49 years in a large European general population sample previously^30^ and cardiac arrhythmias of any type were detected in 17.2% of >10 000 general dentistry patients.^31^ Conceivably, the novelty of the confirmed XLI–cardiac arrhythmia association, the apparently non-impairing and sporadic nature of AHRs in individuals with XLI/female carriers, and the fact that individuals with XLI are typically managed by clinicians without significant expertise in cardiology may explain why high rates of AHRs within XLI populations have not been recognised previously.

So far as we could determine and bearing in mind lack of power, there was no clear relationship between heart rhythm abnormalities and the severity or presence of other features commonly associated with XLI (skin condition, testicular maldescent, neurodevelopmental disorders) implying partially dissociable causes. Our data suggest that, as with idiopathic cardiac arrhythmias, the prevalence of AHR within the Xp22.31 deletion population increases with age. They also indicate that AHRs in Xp22.31 deletion carriers typically resolve quickly and respond well to intervention where this is required. The most commonly reported precipitant within this subpopulation was psychological and/or physiological stress.

Gene-based analyses suggested an aggregate effect of SNPs within *STS* on male (but not female) risk of idiopathic AF. These genetic findings, in combination with: (a) our finding of high rates of AHR in female carriers not affected by XLI and boys unlikely to have been on long-term medication, and (b) medication not being reported as a significant contributor to AHR in males with XLI, provide further evidence against the idea that elevated rates of AHR in Xp22.31 deletion carriers are a secondary consequence of pharmacotherapy but instead support the idea of a biological predisposition to risk.

STS deficiency appears a strong functional candidate for AF risk in Xp22.31 deletion carriers given that *STS* is highly expressed in adult arterial vasculature and at a lower level in the atrial appendage.^32^ Systemic inhibition of the STS enzyme in a cohort of 10 patients with early breast cancer prescreened to exclude a history of cardiac arrhythmia resulted in three grade 2 adverse events (two related to abnormal ECG and one to tachycardia) and one grade 1 adverse event (prolonged QT).^33^ The STS enzyme affects cardiac valve function^34^ and fibrotic pathways,^8 15 35^ and regulates androgen and oestrogen production and the balance between sulfated and non-sulfated steroids such as dehydroepiandrosterone sulfate (DHEAS) and DHEA.^36^ DHEA(S) levels correlate with AF risk in older men in some studies,^12^ and levels of these hormones (and the DHEA:DHEAS ratio) increase upon acute psychosocial stress, with the increase in levels correlating with the stress-induced increase in heart rate.^37^

Despite the UK Biobank and online samples having different ascertainment biases and characteristics, they provided converging evidence that GI problems, asthma and anaemia are the comorbidities most closely associated with AHR in deletion carriers, with GI issues more prominent in male carriers with AHR, and asthma and anaemia more strongly comorbid in female deletion carriers. GI issues are increasingly being recognised as contributors to AF risk via multiple plausible pathways.^38^ Our genetic analyses provide information pertinent to these sex-specific comorbidity effects. Specifically, they implicate *PNPLA4* in asthma and anaemia risk in females only, and *PNPLA4* in GI disorder risk in males only. *PNPLA4* (previously known as GS2 and iPLA2eta) encodes an enzyme with triacylglycerol lipase and acylglycerol transacylase activities,^39^ which may play a role in mitochondrial respiratory chain complex function^40^; PNPLA4 deficiency could feasibly contribute to the lipid metabolism and mitochondrial abnormalities associated with asthma,^41 42^ anaemia^43 44^ and GI disorders.^45 46^ We speculate that loss of *PNPLA4* in female Xp22.31 deletion carriers may predispose to asthma/anaemia while in males loss of *PNPLA4* may predispose to GI issues, and that these medical vulnerabilities may exacerbate any AHR risk incurred as a consequence of STS deficiency. Potentially, effective treatment of GI issues and atopic conditions/anaemia in male and female deletion carriers, respectively, could reduce AHR risk. We did not identify any genetic signatures within Xp22.31 associated with possible downstream consequences of AF including stroke, acute myocardial infarction and dementia; this may be because common variants within Xp22.31 contribute marginally towards AF risk, and AF, in turn, only contributes to the pathogenesis of a relatively small fraction of stroke, acute myocardial infarction and dementia cases.

Work in relevant in vitro (eg, cardiomyocyte/neuronal cultures derived from stem cells from patients with XLI) and in vivo models (eg, *Sts*-deficient mice), in combination with more focused clinical analyses in Xp22.31 deletion carriers guided by the preliminary results presented here, should clarify the physiological, cellular and molecular mechanism(s) through which genetic variants at Xp22.31 affect risk of AF and other relevant medical conditions. In turn, this should lead to better-informed genetic counselling for deletion carriers.

We have shown that individuals with XLI (or female carriers of associated genetic deletions) are strongly in favour of cardiac screening shortly after diagnosis to mitigate long-term health consequences associated with AHRs for them and their offspring. As such, the utility and viability of screening within these populations, and particularly in individuals with comorbid GI disorders, asthma or anaemia where clinical prognosis appears worse,^47 48^ should be investigated. Our results further indicate that routine targeted cardiac screening of both male and female Xp22.31 deletion carriers may be warranted, irrespective of whether they present with XLI-associated phenotypes or not. Such an approach may identify individuals requiring early clinical intervention to mitigate later-life adverse outcomes.

## Data Availability

Data are available as follows: UK Biobank data are available upon application to that resource. Online survey data generated or analysed during this study are included in this published article and its online supplemental files, or are available from https://doi.org/10.17035/d.2022.0230251614.
