# Supplementary material for: Characterising heart rhythm abnormalities associated with Xp22.31 deletion
Source: J Med Genet. 2022 Nov 15;60(7):636–43. doi: 10.1136/jmg-2022-108862 (PMC10359567; doi:10.1136/jmg-2022-108862)
Supplement: Supplementary data [file jmg-2022-108862supp001.pdf]

Supplementary Text 1: Cardiovascular and metabolic conditions investigated as potential risk factors, or comorbidities, for abnormal heart rhythm in our online survey

Coronary artery/heart disease

Pericarditis

Congenital heart disease

Heart failure

Heart attack

Heart murmur

Angina

Stroke

Heart valve disease or malformation

High cholesterol

Anaemia

Hypertension

Cardiomyopathy

Type 1 diabetes

Type 2 diabetes

Rheumatoid arthritis

Inguinal hernia

Pneumonia

Asthma

Gut problem

Lung cancer

Pulmonary embolism

Thyroid gland disorder

Obstructive sleep apnoea

Obesity (BMI>30)

Supplementary Text 2: A vignette regarding our current knowledge of heart rhythm abnormalities in Xp22.31 deletion carriers.

Atrial fibrillation/flutter (AF) is a medical condition characterised by an irregular heart rhythm resulting from disorganised signals to the atria (upper chambers of the heart). The latest evidence suggests that in middle-aged males with XLI, the risk of AF is approximately 4 times greater than in males without XLI (affecting around 10% of males with XLI compared to 2.5% of unaffected males). The scientific literature also describes rare cases of abnormal heart rhythm in young boys with XLI. Individuals with AF are at increased risk of blood clots, and associated disorders including stroke (5x more likely), dementia/cognitive decline (1.5x more likely) and heart failure (3.5x more likely). Stroke, dementia and heart failure can be associated with long-term impairments in mobility and cognitive function, and increased care needs. If identified early, AF can be effectively treated via rate control medication (to lower the heart rate), rhythm control medication (to restore a regular heart rhythm), and/or anti-coagulation (blood clot/stroke prevention) medication, both on a short- and long-term basis. Individuals may be monitored for AF via an electrocardiogram (ECG) (wires attached to the chest, routinely undertaken at local doctors), blood pressure monitoring (using a cuff placed around the arm) and cardiovascular examinations (examining any external physical indicators e.g. skin discolouration, eyes) via hospital appointments from early life, and appropriate interventions administered.

Supplementary Table 1. Demographic variables in individuals with and without self- or parent-reported abnormal heart rhythms

**Adult males with XLI (n=43)**			
	**Age (yrs)**	**Country of residence**	**Ethnicity**
**With abnormal heart rhythm (AHR)**	51.5 (95%CI:42.6-60.6)	UK:47%<br>USA:27%<br>Other:26%	White European: 100%<br>Other: 0%
**Without abnormal heart rhythm (AHR)**	46.2 (95%CI:41.1-51.2)	UK:54%<br>USA:36%<br>Other:10%	White European:86%<br>Other: 14%
	t[40]=1.09, p=0.28	$\chi^2_{[2]}$=4.67, p=0.10	$\chi^2_{[1]}$=2.36, p=0.12
**Adult female carriers (n=79)**			
	**Age (yrs)**	**Country of residence**	**Ethnicity**
**With abnormal heart rhythm (AHR)**	43.7 (95%CI: 39.1-48.2)	UK:35%<br>USA:40%<br>Other:25%	White European: 90%<br>Other: 10%
**Without abnormal heart rhythm (AHR)**	40.9 (95%CI: 38.2=43.6)	UK:37%<br>USA:37%<br>Other:26%	White European: 88%<br>Other: 12%
	t[57]=0.57, p=0.57	$\chi^2_{[2]}$=0.51, p=0.78	$\chi^2_{[1]}$=0.77, p=0.38
**Boys with XLI (n=69)**			
	**Age (yrs)**	**Country of residence**	**Ethnicity**
**With abnormal heart rhythm (AHR)**	8.9 (95%CI: 6.3-11.4)	UK:40%<br>USA:40%<br>Other:20%	White European: 80%<br>Other: 20%
**Without abnormal heart rhythm (AHR)**	7.5 (95%CI: 6.1-9.0)	UK:41%<br>USA:35%<br>Other:24%	White European: 91%<br>Other: 9%
	t[67]=0.40, p=0.69	$\chi^2_{[2]}$=0.16, p=0.92	$\chi^2_{[1]}$=1.32, p=0.25

Supplementary Table 2A. Individuals with or without abnormal heart rhythms (AHR) diagnosed with cardiovascular and metabolic conditions

Condition	Yes/No	XLI males with AHR (n=15)	XLI males without AHR (n=24)	XLI females with AHR (n=20)	XLI females without AHR (n=51)	XLI boys with AHR (n=15)	XLI boys without AHR (n=48)	All males with AHR (n=30)	All males without AHR (n=72)	All participants with AHR (n=50)	All participants without AHR (n=123)
Coronary artery/heart disease	Yes	0	0	1	0	0	0	0	0	1	0
	No	15	24	19	51	15	48	30	72	49	123
Pericarditis	Yes	0	0	0	0	0	0	0	0	0	0
	No	15	24	20	51	15	48	30	72	50	123
Congenital heart disease	Yes	0	0	1	0	1	0	1	0	2	0
	No	15	24	19	51	14	48	29	72	48	123
Heart failure	Yes	0	0	0	0	0	0	0	0	0	0
	No	15	24	20	51	15	48	30	72	50	123
Heart attack	Yes	0	1	0	0	0	0	0	1	0	1
	No	15	23	20	51	15	48	30	71	50	122
Heart murmur	Yes	0	0	4	0	2	7	2	7	6	7
	No	15	24	16	51	13	41	28	65	44	116
Angina	Yes	1	0	0	0	0	0	1	0	1	0
	No	14	24	20	51	15	48	29	72	49	123
Stroke	Yes	0	0	0	0	0	0	0	0	0	0
	No	15	24	20	51	15	48	30	72	50	123
Heart valve disease or malformation	Yes	1	0	2	1	1	0	2	0	4	1
	No	14	24	18	50	14	48	28	72	46	122

**High cholesterol**	**Yes**	4	3	3	7	0	0	4	3	7	10
	**No**	11	21	17	44	15	48	26	69	43	113
**Anaemia**	**Yes**	0	0	8	6	1	2	1	2	9	8
	**No**	15	24	12	45	14	46	29	70	41	115
**Hypertension**	**Yes**	5	4	4	10	0	0	5	4	9	14
	**No**	10	20	16	41	15	48	25	68	41	109
**Cardiomyopathy**	**Yes**	0	0	1	0	0	0	0	0	1	0
	**No**	15	24	19	51	15	48	30	72	49	123
**Type I diabetes**	**Yes**	1	0	0	0	0	0	1	0	1	0
	**No**	14	24	20	51	15	48	29	72	49	123
**Type II diabetes**	**Yes**	1	2	2	3	0	0	1	2	3	5
	**No**	14	22	18	48	15	48	29	70	47	118
**Rheumatoid arthritis**	**Yes**	1	0	1	1	0	0	1	0	2	1
	**No**	14	24	19	50	15	48	29	72	48	122
**Inguinal hernia**	**Yes**	1	0	1	3	0	0	1	0	2	3
	**No**	14	24	19	48	15	48	29	72	48	120
**Pneumonia**	**Yes**	2	3	2	3	2	2	4	5	6	8
	**No**	13	21	18	48	13	46	26	67	44	115
**Asthma**	**Yes**	4	5	7	5	4	6	8	11	15	16
	**No**	11	19	13	46	11	42	22	61	35	107
**Gut problem**	**Yes**	4	0	5	4	1	2	5	2	10	6
	**No**	11	24	15	47	14	46	25	70	40	117

**Lung cancer**	**Yes**	0	0	0	0	0	0	0	0	0	0
	**No**	15	24	20	51	15	48	30	72	50	123
**Pulmonary embolism**	**Yes**	0	0	0	2	0	0	0	0	0	2
	**No**	15	24	20	49	15	48	30	72	50	121
**Thyroid gland disorder**	**Yes**	2	1	2	6	1	0	3	1	5	7
	**No**	13	23	18	45	14	48	27	71	45	116
**Obstructive sleep apnoea**	**Yes**	1	3	3	4	0	1	1	4	4	8
	**No**	14	21	17	47	15	47	29	68	46	115
**Obesity (BMI>30)**	**Yes**	2	2	4	9	0	3	2	5	6	14
	**No**	13	22	16	42	15	45	28	67	44	109

Supplementary Table 2B Associated statistical analysis for **Supp Table 2A (**two-tailed p-values from Fisher Exact Test) *p≤0.05, **p≤0.005

**Condition**	**XLI adult males (AHR vs. no AHR)**	**Female carriers (AHR vs. no AHR)**	**XLI boys (AHR vs. no AHR)**	**All males (AHR vs. no AHR)**	**All participants (AHR vs. no AHR)**
**Coronary artery/heart disease**	1.000	0.282	1.000	1.000	0.289
**Pericarditis**	1.000	1.000	1.000	1.000	1.000
**Congenital heart disease**	1.000	0.282	0.238	0.294	0.082
**Heart failure**	1.000	1.000	1.000	1.000	1.000
**Heart attack**	1.000	1.000	1.000	1.000	1.000
**Heart murmur**	1.000	0.005**	1.000	0.723	0.202
**Angina**	0.385	1.000	1.000	0.294	0.289
**Stroke**	1.000	1.000	1.000	1.000	1.000

**Heart valve disease or malformation**	0.385	0.189	0.238	0.084	0.025*
**High cholesterol**	0.396	1.000	1.000	0.190	0.265
**Anaemia**	1.000	0.011*	1.000	1.000	0.027*
**Hypertension**	0.266	1.000	1.000	0.119	0.322
**Cardiomyopathy**	1.000	0.282	1.000	1.000	0.289
**Type I diabetes**	0.385	1.000	1.000	0.294	0.289
**Type II diabetes**	1.000	0.616	1.000	1.000	0.692
**Rheumatoid arthritis**	0.385	1.000	1.000	0.294	0.201
**Inguinal hernia**	0.385	1.000	1.000	0.294	0.627
**Pneumonia**	1.000	0.616	0.238	0.443	0.356
**Asthma**	0.711	0.017*	0.231	0.263	0.010*
**Gut problem**	0.017*	0.105	1.000	0.022*	0.004**
**Lung cancer**	1.000	1.000	1.000	1.000	1.000
**Pulmonary embolism**	1.000	1.000	1.000	1.000	1.000
**Thyroid gland disorder**	0.547	1.000	0.238	0.075	0.332
**Obstructive sleep apnoea**	0.648	0.394	1.000	1.000	0.746
**Obesity (BMI>30)**	1.000	1.000	0.574	1.000	1.000

Supplementary Table 3. Percentage of individuals with XLI (or female carriers) reporting involvement of precipitating factor in onset of AHR episodes

Precipitating factor	Adults males with XLI and AHR (n=12)	Female carriers with AHR (n=20)	Boys with XLI and AHR (n=14)
Stress	42	45	14
Medication	8	0	7
Caffeine consumption (tea, coffee, energy drinks	33	25	7
Smoking	8	0	0
Postural change (e.g. moving from sitting down to standing up)	17	25	7
Sleep disturbance	33	15	7
Infection	8	10	14
Exercise	17	35	14
Increased body temperature e.g. due to inability to sweat	17	15	29
No obvious cause	42	25	50
Other	17	30	14

Supplementary Table 4. Percentage of individuals with XLI (or female carriers) reporting times of onset of AHR episodes

Time of onset	Adults males with XLI and AHR (n=12)	Female carriers with AHR (n=12)	Boys with XLI and AHR (n=7)
Whilst sleeping/in the night	57	42	57
Early morning, shortly after waking	57	8	14
During the day	57	75	86
Late at night, just before sleeping	43	58	29

Supplementary Table 5. A gene-based analysis of SNPs associated with stroke in the consensus Xp22.31 deletion interval in males (3,713 cases vs. 191,861 controls) and females (3,128 cases vs. 164,873 controls) from the UK Biobank. NSNPs= the number of SNPs in the data annotated to the gene; NParam = the number of relevant parameters used in the model (essentially the number of independent SNPs in the gene).

Gene	Start (bp)	Stop (bp)	Males			Females		
			NSNPs	NParam	P-value	NSNPs	NParam	P-value
VCX3A	6451659	6453159	5	3	0.651	5	3	0.859
HDHD1	6966961	7066231	225	12	0.666	223	13	0.208
STS	7065298	7272682	343	20	0.839	336	20	0.115
VCX	7810303	7812184	4	2	0.929	3	2	0.099
PNPLA4	7866804	7895780	39	6	0.084	39	6	0.061
VCX2	8137985	8139308	11	4	0.813	8	3	0.494

Supplementary Table 6. A gene-based analysis of SNPs associated with acute myocardial infarction in the consensus Xp22.31 deletion interval in males (12,186 cases vs. 155,869 controls) and females (3,927 cases vs. 191,711 controls) from the UK Biobank. NSNPs= the number of SNPs in the data annotated to the gene; NParam = the number of relevant parameters used in the model (essentially the number of independent SNPs in the gene).

Gene	Start (bp)	Stop (bp)	Males			Females		
			NSNPs	NParam	P-value	NSNPs	NParam	P-value
VCX3A	6451659	6453159	5	3	0.793	5	3	0.146
HDHD1	6966961	7066231	225	12	0.903	223	13	0.746
STS	7065298	7272682	343	20	0.830	336	20	0.777
VCX	7810303	7812184	4	2	0.483	3	2	0.390
PNPLA4	7866804	7895780	39	6	0.557	39	6	0.795
VCX2	8137985	8139308	11	4	0.318	8	3	0.171

Supplementary Table 7. A gene-based analysis of SNPs associated with dementia in the consensus Xp22.31 deletion interval in males (2,238 cases vs. 165,817 controls) and females (1,892 cases vs. 193,746 controls) from the UK Biobank. NSNPs= the number of SNPs in the data annotated to the gene; NParam = the number of relevant parameters used in the model (essentially the number of independent SNPs in the gene).

Gene	Start (bp)	Stop (bp)	Males			Females		
			NSNPs	NParam	P-value	NSNPs	NParam	P-value
VCX3A	6451659	6453159	5	3	0.228	5	3	0.729
HDHD1	6966961	7066231	225	12	0.843	223	13	0.537
STS	7065298	7272682	343	20	0.865	336	20	0.429
VCX	7810303	7812184	4	2	0.893	3	2	0.433
PNPLA4	7866804	7895780	39	6	0.690	39	6	0.839
VCX2	8137985	8139308	11	4	0.453	8	3	0.390

Supplementary Table 8. A gene-based analysis of SNPs associated with asthma in the consensus Xp22.31 deletion interval in males (22,231 cases vs. 145,824 controls) and females (29,206 cases vs. 166,432 controls) from the UK Biobank. NSNPs= the number of SNPs in the data annotated to the gene; NParam = the number of relevant parameters used in the model (essentially the number of independent SNPs in the gene). *p<0.05

Gene	Start (bp)	Stop (bp)	Males			Females		
			NSNPs	NParam	P-value	NSNPs	NParam	P-value
VCX3A	6451659	6453159	5	3	0.170	5	3	0.484
HDHD1	6966961	7066231	225	12	0.994	223	13	0.909
STS	7065298	7272682	343	20	0.732	336	20	0.880
VCX	7810303	7812184	4	2	0.207	3	2	0.873
PNPLA4	7866804	7895780	39	6	0.196	39	6	0.040*
VCX2	8137985	8139308	11	4	0.150	8	3	0.596

Supplementary Table 9. A gene-based analysis of SNPs associated with anaemia in the consensus Xp22.31 deletion interval in males (7,958 cases vs. 160,097 controls) and females (14,015 cases vs. 181,623 controls) from the UK Biobank. NSNPs= the number of SNPs in the data annotated to the gene; NParam = the number of relevant parameters used in the model (essentially the number of independent SNPs in the gene). *p<0.05

Gene	Start (bp)	Stop (bp)	Males			Females		
			NSNPs	NParam	P-value	NSNPs	NParam	P-value
VCX3A	6451659	6453159	5	3	0.438	5	3	0.551
HDHD1	6966961	7066231	225	12	0.210	223	13	0.981
STS	7065298	7272682	343	20	0.242	336	20	0.971
VCX	7810303	7812184	4	2	0.165	3	2	0.623
PNPLA4	7866804	7895780	39	6	0.584	39	6	0.013*
VCX2	8137985	8139308	11	4	0.120	8	3	0.680

Supplementary Table 10. A gene-based analysis of SNPs associated with gastrointestinal disorders in the consensus Xp22.31 deletion interval in males (42,680 cases vs. 125,375 controls) and females (56,842 cases vs. 138,796 controls) from the UK Biobank. NSNPs= the number of SNPs in the data annotated to the gene; NParam = the number of relevant parameters used in the model (essentially the number of independent SNPs in the gene). *p<0.05

Gene	Start (bp)	Stop (bp)	Males			Females		
			NSNPs	NParam	P-value	NSNPs	NParam	P-value
VCX3A	6451659	6453159	5	3	0.021*	5	3	0.650
HDHD1	6966961	7066231	225	12	0.553	223	13	0.344
STS	7065298	7272682	343	20	0.610	336	20	0.296
VCX	7810303	7812184	4	2	0.044*	3	2	0.283
PNPLA4	7866804	7895780	39	6	0.022*	39	6	0.470
VCX2	8137985	8139308	11	4	0.018*	8	3	0.113
